# The frontal aslant tract and its role in executive functions: a quantitative tractography study in glioma patients

**DOI:** 10.1007/s11682-021-00581-x

**Published:** 2021-10-30

**Authors:** Maud J. F. Landers, Stephan P. L. Meesters, Martine van Zandvoort, Wouter de Baene, Geert-Jan M. Rutten

**Affiliations:** 1 Department of Neurosurgery, Elisabeth-Tweesteden Hospital, Tilburg, The Netherlands; 2 Department of Neurology & Neurosurgery, University Medical Centre Utrecht, Utrecht, The Netherlands; 3 Department of Mathematics and Computer Science, Eindhoven University of Technology, Eindhoven, The Netherlands; 4 Department of Cognitive Neuropsychology, Tilburg University, Tilburg, The Netherlands

**Keywords:** Frontal aslant tract, Lesion-symptom study, Tractography, Executive functions, Glioma patients

## Abstract

Focal white matter lesions can cause cognitive impairments due to disconnections within or between networks. There is some preliminary evidence that there are specific hubs and fiber pathways that should be spared during surgery to retain cognitive performance. A tract potentially involved in important higher-level cognitive processes is the frontal aslant tract. It roughly connects the posterior parts of the inferior frontal gyrus and the superior frontal gyrus. Functionally, the left frontal aslant tract has been associated with speech and the right tract with executive functions. However, there currently is insufficient knowledge about the right frontal aslant tract’s exact functional importance. The aim of this study was to investigate the role of the right frontal aslant tract in executive functions via a lesion-symptom approach. We retrospectively examined 72 patients with frontal glial tumors and correlated measures from tractography (distance between tract and tumor, and structural integrity of the tract) with cognitive test performances. The results indicated involvement of the right frontal aslant tract in shifting attention and letter fluency. This involvement was not found for the left tract. Although this study was exploratory, these converging findings contribute to a better understanding of the functional frontal subcortical anatomy. Shifting attention and letter fluency are important for healthy cognitive functioning, and when impaired they may greatly influence a patient’s wellbeing. Further research is needed to assess whether or not damage to the right frontal aslant tract causes permanent cognitive impairments, and consequently identifies this tract as a critical pathway that should be taken into account during neurosurgical procedures.

## Introduction

A major goal in brain tumor surgery is to understand the functionality of peritumoral tissue in order to safely optimize the resection. Over the past decade, the functional importance of white matter pathways has been increasingly acknowledged in surgical planning. MRI-based tractography and intraoperative electrical stimulation mapping have become established tools to identify fiber pathways for sensorimotor, visual and language functions (Henderson et al., 2020; Mandonnet et al., 2017; Ruttenet al., 2014). For higher cognitive functions, such as executive functioning, there are no such protocols yet. Although their nomenclature is widely discussed, executive functions can be described as ‘a set of general-purpose control mechanisms that regulate the dynamics of human cognition and action’ (Miyake & Friedman, 2012). Executive functions (including inhibition, working memory, planning, monitoring) are often linked to the frontal lobe, and their impairment likely has significant negative implications for normal social and professional life (Duffau & Mandonnet, 2013; Mischel et al., 2011; Moffitt et al., 2011).

The study of patients with brain lesions has shed important light on the functional architecture of the brain, by providing unique insight into the indispensability of certain brain structures (Damasio & Damasio, 1989; Lus et al., 2011; Sanai & Berger, 2018). As such, lesion-symptom studies remain a valuable adjunct to studies that use contemporary neuroimaging techniques (Adolphs, 2016; Bates et al., 2003; Lawes et al., 2008; Mah et al., 2014). There is some preliminary evidence that focal white matter lesions can induce cognitive impairments due to disconnections within and between networks, and that there are fiber pathways that should be spared during surgery to retain pre-surgical level of cognitive performance (Leclercq et al., 2011; Mandonnet et al., 2017). A relatively newly discovered white matter pathway is the frontal aslant tract (FAT). The FAT connects pars opercularis and pars triangularis of the inferior frontal gyrus to the pre-Supplementary Motor Area (pre-SMA) and the Supplementary Motor Area (SMA) of the superior frontal gyrus (Aron et al., 2007; Dick et al., 2014). It was first described in 2007 and named ‘aslant tract’ several years later due to its oblique course in the frontal white matter (Catani et al., 2012; Dick et al., 2019). The connectivity of this tract was visualized with diffusion-weighted magnetic resonance imaging (DW-MRI) and has been verified with anatomical post-mortem evidence (Bozkurt et al., 2016; Catani et al., 2013; Dick et al., 2019; Mandelli et al., 2014; Vergani et al., 2014).

Functionally, the FAT has been linked to speech, language, motor-action control and executive functions (Dick et al., 2019), with some level of lateralization. The left FAT has been strongly associated with motor-speech functions and, to a lesser extent, with language networks involved in lexical, phonological and syntactic processing (Broce et al., 2015; Catani et al., 2013; Dick et al., 2019; Hickok & Poeppel, 2007; Kinoshita et al., 2015; Mandelli et al., 2014). Part of this evidence comes from intraoperative stimulation in brain tumor patients that underwent awake craniotomies, in whom speech arrest occurred upon stimulation of the left FAT (Corrivetti et al., 2019; Rutten, 2015; Szelényi et al., 2010; Vassal et al., 2014). Additional evidence comes from a lesion-symptom study, in which the distance between the resection cavity of a frontal glioma and the FAT was positively correlated with speech initiation and verbal fluency (Kinoshita et al., 2015), and from patients with non-fluent aphasia, in whom a loss of fibers of the FAT resulted in problems with phonological processing and articulation (Ille et al., 2018).

In contrast to the left FAT, much less in known about the functionality of the right FAT. Studies that examined the areas that are connected by the right FAT suggest that it is involved in executive functions (Aron et al., 2014; Dick et al., 2019; Erika-Florence et al., 2014). These areas play a role in inhibitory control, conflict monitoring and working memory (Aron et al., 2014; Erika-Florence et al., 2014; Varriano et al., 2018). All three functions are thought to be involved in top-down executive control (Aron et al., 2007; Postle et al., 1999). Three studies supported that the inhibitory control network has a right lateralized architecture, as in inhibition of manual actions (Budisavljevic et al., 2017; Wiecki & Frank, 2013) and in stopping and resolving conflict (Aron et al., 2016). While the currently available literature suggests that the right FAT is involved in functions that require top-down executive control, evidence is still limited.

The aim of this study was to explore the role of the right FAT in executive functions via a lesion-symptom approach. This was investigated in patients with frontal brain tumors, assuming that the presence of a tumor can lead to a dysfunction of the right FAT, either directly (via infiltration) or indirectly (via mass effect or oedema) (Duffau et al., 2008; Jellison et al., 2004; Mormina et al., 2015). We hypothesized that executive control (as measured with cognitive tests) will be negatively affected by the following parameters: (i) spatial proximity of the tumor to the FAT in the right hemisphere (but no such relation in the left hemisphere) and (ii) disturbed structural integrity of the right FAT as measured with DWI.

## Methods

### Patient selection

We retrospectively analyzed patients with low- and high-grade gliomas, from whom DW-MRI was acquired in the week before surgery (between 4 and 1 days prior to surgery), according to the standard presurgical functional imaging protocol of the Elisabeth-TweeSteden hospital (Tilburg, the Netherlands). These patients underwent brain surgery in the period between November 2010 and March 2019 and were eligible for the current study if the tumor was located in the frontal lobe and they also had received a computerized neuropsychological assessment in the week before surgery (Rijnen et al., 2017). Exclusion criteria were age below 18 years, a recent history of other major medical illnesses in the past year prior to surgery, previous craniotomy, severe psychiatric or neurological disorders in the past two years and lack of basic Dutch language skills. This study was approved by the local ethics committee (NW2020-32, METC Brabant, The Netherlands) and all patients gave written informed consent to participate and for the use of data for research purposes.

### Measures and procedure

#### DWI Tractography by means of Constrained Spherical Deconvolution (CSD)

**Fig. 1:**
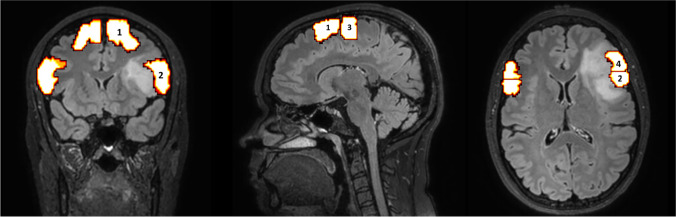
Coronal, sagittal and axial slice of one of the subjects presenting the ROIs (in orange) with pre-SMA (1) and SMA (3) as seed locations and pars opercularis (2) and pars triangularis (4) as target locations. A = anterior S = superior P = posterior R = right L = left

All DWI scans were acquired using a Philips Achieva 3T MRI-scanner (b=1500, 50 diffusion weighting directions, 6 b=0 images, 2 mm isotropic voxel size). Tractography was performed using the MRTrix software package (Tournier et al., 2012). DW-MRI data was pre-processed using the MRTrix script *dwipreproc*. Probabilistic tractography was performed using the constrained spherical deconvolution-based iFOD2 method with *tckgen*. To enable tractography of the FAT, estimated regions of interest (ROIs) were automatically determined via registration to anatomical atlases (Fig. 1) (see *Coregistration*). Pre-SMA and SMA functioned as seed locations and were identified using an atlas from Neubert Cingulate Orbito Frontal Parcellation (Neubert et al., 2014). Pars opercularis and pars triangularis functioned as target locations and were identified using an atlas from Tailarach (Lancaster et al., 2000).

All ROIs were verified by at least two medical professionals, whom defined boundaries based on previous anatomical studies (Lima et al., 2016; Petrides, 2006; Picard & Strick, 1996). In short we identified the following: as anterior boundary of the pre-SMA: a vertical virtual plane passing through the genu of corpus callosum; as posterior boundary of the pre-SMA and anterior boundary of the SMA: a vertical plane passing through the anterior commissure perpendicular to the AC-PC; as posterior boundary of the SMA: the sulcus precentralis; as anterior boundary of the pars triangularis: the ramus horizontalis; as posterior boundary of the pars triangularis and anterior boundary of the pars opercularis: the ramus ascendens; as posterior boundary of the pars opercularis: the sulcus precentralis.

#### Tumor segmentation

Tumor segmentations were conducted semi-automatically using active contours available in ITK-SNAP (Yushkevich et al., 2006). This technique involves some manual assistance to set tumor margins and then automatically segments the tumor. In case of high-grade gliomas T1-weighted contrast images were used to identify tumor margins. In case of non-enhancing gliomas, FLAIR images were used to delineate tumor and surrounding tissue. All segmentations were verified by at least two medical professionals.

#### Coregistration

**Fig. 2:**
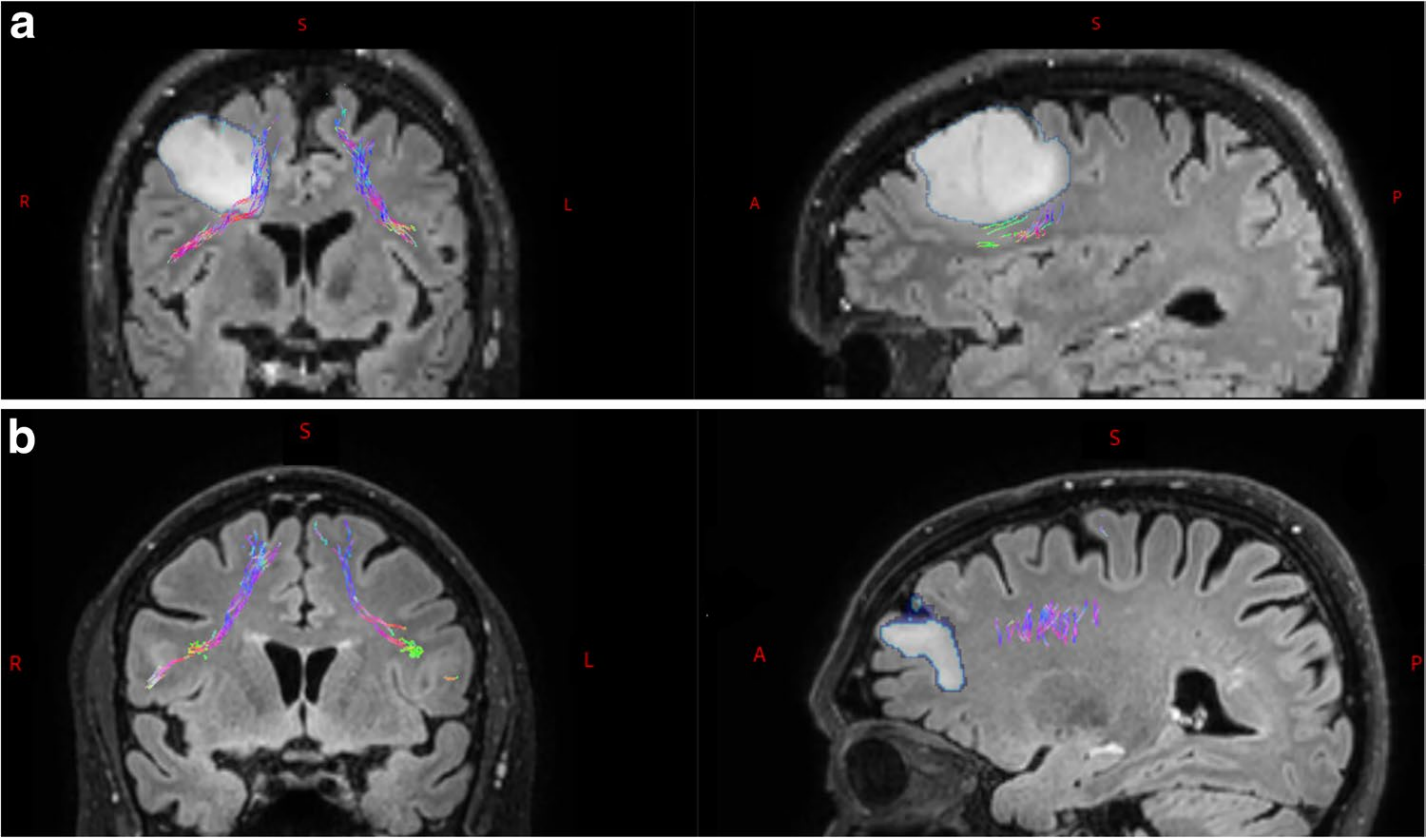
**A** Coronal and sagittal slice of one subject presenting the relation between the tumor (outlined in light blue) and the right FAT (in different colors representing the direction). In this example the tumor adjoins the FAT, with a mean minimal distance of 5mm. A = anterior S = superior P = posterior R = right L = left. **B** Coronal and sagittal slice of one subject presenting the relation between the tumor (outlined in light blue) and the right FAT (in different colors representing the direction). In this example the tumor does not adjoin the FAT, with a mean minimal distance of 23mm

For further data processing, all images (T1/T2/Flair, DWI, tractography ROIs and tumor segmentations) were transferred into the same diffusion-weighted MRI space (Fig. 2A and B) using NiftyReg affine coregistration (Clayden et al., 2019).

#### Mean minimal distance

The spatial relationship between ipsilateral FAT and tumor was investigated by calculating a mean distance measure. An algorithm calculated the shortest distance between each fiber of the tract and the most nearby tumor voxel. The average of the shortest distances of all fibers (in millimeters) was named the mean minimal distance (Min-mean). This algorithm was implemented in Wolfram Mathematica 12 (Wolfram Research, 2019).

#### Fractional anisotropy and mean diffusivity

To investigate the structural integrity of the FAT, two measures were calculated using *tcksample*, after calculating tensors with the MRtrix method *dwi2tensor.* This generated values for each voxel of the FAT, which were averaged, resulting in average fractional anisotropy and mean diffusivity. Both are well-known measures of axonal integrity (Mormina et al., 2015). Fractional anisotropy (FA) is a measure that reflects the coherent directionality of water diffusion, or anisotropy, caused by the constriction of water molecules across the walls of axons. In the case of organized white matter tracts (i.e. the axons are well aligned) the FA will be close to 1, while in the case of disorganized fiber structures or the absence of axons the FA will be close to 0 (De Erausquin & Alba-Ferrara, 2013; Jones et al., 2013). Mean diffusivity (MD) is an isotropic measure that reflects water diffusion in each direction, being the inverse of membrane density. Isotropic measures can increase due to any disease process that affects barriers, and they are highest in cerebrospinal fluid (Alexander et al., 2007).

#### Neuropsychological assessment

Neuropsychological assessment was performed in the week prior to surgery (between 4 and 1 days prior to surgery) using six tests, administered as part of standard clinical care. Five tests were recorded using the Dutch version of a computerized neuropsychological test battery (Appendix Table 6). The tests included finger tapping, symbol digit coding, shifting attention, continuous performance, and Stroop interference. Z-scores were calculated to adjust for age, sex and education level based on a Dutch normative control sample (Rijnen et al., 2017). In addition, letter fluency was also assessed, using a Dutch version of the Controlled Oral Word Association (COWAT) letter fluency test. Z-scores for the latter test were adjusted for education level only, as age and sex do not significantly influence the test scores (Schmand et al., 2008). Scores were reversed for continuous performance and Stroop interference, so that for all tests lower z-scores indicate worse performance. Statistical analyses were performed using SPSS 24.0 (Corp., Released 2016).

### Statistical analysis

#### Descriptive statistics

Descriptive analyses were performed for the following participant characteristics: age, sex, level of education, affected hemisphere, tumor type, tumor volume and handedness.

#### Spearman’s rank-order correlation

The relationship between the distance measure and cognitive outcome measures was explored by conducting a Spearman’s correlation analysis, because variables were not normally distributed. The relationship between the structural integrity measures (FA, MD) and cognitive outcome measures was explored by conducting an additional Spearman’s correlation analysis. For both analyses assumptions for Spearman’s correlation were first evaluated. Data was quality controlled for outliers by computing Cook’s distances (Cook & Weisberg, 1982). To control for multiple statistical testing, corrected alpha values were calculated and set against *p*-values, using the Benjamini-Hochberg procedure to reduce the false discovery rate (Benjamini & Hochberg, 1995). False discovery rates were set at 0.1 given the exploratory character of this study (Glickman et al., 2014).

#### Linear regression

To assess whether the distance measure and structural integrity measures are prognostic factors for performance on different cognitive tests, linear regressions were run for each of them. For all three measures assumptions for linear regression were evaluated. Tumor grade, tumor volume and handedness were included in a base model to test in a multivariable model if addition of a distance or structural integrity measure to the base model accounted for a significant effect on cognitive test performance.

## Results

### Descriptive statistics

In total, 182 glioma patients received a DW-MRI-scan and neuropsychological assessment before surgery in the period November 2010 and March 2019. In 99 of these 182 cases, the tumor was located in the frontal lobe, 16 of which were not eligible based on the exclusion criteria (12 underwent brain surgery in the past, 3 had major medical comorbidities and 1 was incompetent in Dutch). For 11 of the remaining participants, neuropsychological assessment was not completed or considered unreliable due to interfering factors. This resulted in a total of 72 participants. Table 1 provides an overview of the patient characteristics. No significant differences were found between the different categories for neither of the following characteristics on cognitive test performance: sex, level of education, affected hemisphere, tumor type, tumor volume median and handedness (with smallest level of significance *p* = .095). Letter fluency was not assessed in 22 of 72 participants, resulting in a total of 50 participants with letter fluency scores. Participant characteristics from the latter group did not differ significantly from the overall group (*n* = 72). Table 2 shows an overview of distance and structural integrity measures for both the affected and non-affected hemisphere.

**Table 1:** Patient characteristics (n=72)

Age mean (SD; range) in years		42.0 (13.0; 20-67)	
Sex (*n*)	Male Female	36 36	50 % 50 %
Level of education (*n*)^1^	Low Middle High	9 27 36	12.5 % 37.5 % 50 %
Affected hemisphere (*n*)	Right Left	32 40	44.4 % 55.6 %
Tumor type (*n*)^2^	Grade 2 Grade 3 Grade 4	49 10 13	68 % 13.9 % 18.1 %
Tumor volume median (Q1;Q3)^3^ in cm3	Right Left	32.0 (18.2; 45.0) 37.7 (24.4; 83.8)	
Handedness (*n*)	Right Left	60 12	83.3 % 16.7 %

^1^Education classified according to Dutch coding system of Verhage and categorized into Low: Verhage 1-4, Middle: Verhage 5 and High: Verhage 6 -7 (Verhage, 1964)

^2^Tumor type classified according to the WHO grading system of central nervous system tumors (Louis et al. 2016); ^3^Quartile 1, median of the lower half of the dataset; Quartile 3, median of the upper half of the dataset

**Table 2:** Distance and structural measures (n=72)

	Right hemisphere FAT		Left hemisphere FAT	
	Tumor LH^1^ *n*= 40	Tumor RH^2^ *n*= 32	Tumor LH^1^ *n*= 40	Tumor RH^2^ *n*= 32
Distance median^3^ (Q1;Q3)^4^ Minimal mean	n/a	5.70 (3.93; 12.34)	5.68 (3.93; 9.03)	n/a
Structural mean (SD) FA MD ^1 × 10e−3^	0.43 (0.04) 0.77 (0.09)	0.38 (0.07) 0.86 (0.15)	0.37 (0.08) 0.90 (0.16)	0.42 (0.03) 0.78 (0.07)

^1^ Tumor located in left hemisphere; ^2^ Tumor located in right hemisphere

^3^Distance in millimeters. Distance to tumor in non-affected hemisphere irrelevant. ^4^Quartile 1, median of the lower half of the dataset; Quartile 3, median of the upper half of the dataset

### Correlation analyses distance measure and structural integrity measures

Preliminary analyses including visual inspection of scatterplots showed the relationships of both the distance measure and the structural integrity measures with cognitive test performance to be not perfectly monotonic, but assumptions for Spearman’s rank-order correlation were met. Table 3 presents the results of the Spearman’s correlation analyses. The unadjusted *p*-values are presented in bold if they remained significant after correction for multiple comparisons.

**Table 3:** Spearman’s rank-order correlation

	Minimal mean distance:			Structural integrity measures:		
			MD	FA		
Cognitive tests:	Right (*n=*32*)* rho ***p*** <	Left (*n=*40*)* rho ***p*** <	Right (*n=*32*)* rho ***p*** <	Left (*n*=40) rho ***p*** <	Right (*n=*32*)* rho ***p*** <	Left (*n*=40) rho ***p*** <
Finger tapping	0.183 0.315	-0.023 0.890	0.297 0.098	-0.062 0.704	-0.292 0.105	0.005 0.978
Symbol digit coding	-0.063 0.730	0.175 0.280	0.397* 0.024	0.036 0.825	-0.407* ***0.021***	-0.084 0.605
Shifting attention	0.453** 0.009	0.118 0.467	0.328 0.066	0.182 0.261	-0.365* 0.040	-0.129 0.426
Continuous performance	0.351* 0.049	0.030 0.855	0.344 0.054	-0.087 0.593	-0.453** ***0.009***	0.170 0.294
Stroop Interference	-0.183 0.371	0.177 0.274	-0.006 0.973	0.185 0.252	0.005 0.979	-0.267 0.096
Letter fluency^1^	0.489* 0.025	0.177 0.349	0.422 0.056	0.029 0.878	-0.501* ***0.021***	-0.025 0.897

** meaning *p* < .01; * meaning *p* < .05; In bold *p* < BH-corrected alpha of 0.1; In italics *p* < BH-corrected alpha of 0.05

^1^ Data missing right for *n*=12 and left for *n*=10

For shifting attention and letter fluency, statistically significant, positive correlations were found for the minimal mean distance in right-sided tumors (*p*s < BH-corrected alpha 0.1). For symbol digit coding, shifting attention, continuous performance and letter fluency, statistically significant negative correlations were found for MD in right-sided tumors (*ps* < BH-corrected alpha 0.1). For FA in right-sided tumors and for FA and MD in left-sided tumors no statistically significant correlations were found for any of the tests.

### Linear regression for distance

Preliminary analyses included visual inspection of scatterplots and showed data to be non-normally distributed for all variables. To deal with this non-normality, bootstrapping based on 1000 samples was performed, as this generates bootstrapped p-values and confidence intervals based on percentiles instead of standard errors, which can be used for significance testing. All other assumptions were met. The results of the linear regression analyses are presented in Table 4, presenting full details of the base model and the multivariable model.

**Table 4:** Linear regression using bootstrapping

		Distance measures:			
Cognitive tests:		Min-Mean right (*n=*32*)*		Min-Mean left (*n=*40*)*	
Finger tapping	Base model^a^	*F*(3, 28) = 2.824, *p* = .057, *R*^*2*^ = 0.232		* F*(3, 36) = 0.984, *p* = .411, *R*^*2*^ = 0.076	
	B (95 %CI)^b^ Fchange (df)^b^ R^2^ ^b^	0.023 (-0.086 to 0.126) 0.300 (1, 27) 0.241		-0.003 (-0.127 to 0.140) 0.003 (1, 35) 0.076	
Symbol digit coding	Base model^a^	*F*(3, 28) = 1.560, *p* = .221, *R*^*2*^ = 0.143		* F*(3, 36) = 0.272, *p* = .845, *R*^*2*^ = 0.022	
	B (95 %CI)^b^ Fchange (df)^b^ R^2^ ^b^	-0.017 (-0.072 to 0.033) 0.347 (1, 27) 0.154		0.030 (-0.036 to 0.095) 0.567 (1, 35) 0.038	
Shifting attention	Base model^a^	*F*(3, 28) = 0.767, *p* = .522, *R*^*2*^ = 0.076		* F*(3, 36) = 1.317, *p* = .284, *R*^*2*^ = 0.099	
	B (95 %CI)^b^ Fchange (df)^b^ R^2^ ^b^	0.061* (0.025 to 0.109) 4.589 (1, 27) 0.210		0.040 (-0.045 to 0.130) 0.521 (1, 35) 0.112	
Continuous performance	Base model^a^	*F*(3, 28) = 1.343, *p* = .280, *R*^*2*^ = 0.126		* F*(3, 36) = 0.389, *p* = .762, *R*^*2*^ = 0.031	
	B (95 %CI)^b^ Fchange (df)^b^ R^2^ ^b^	0.067 (-0.008 to 0.178) 2.067 (1, 27) 0.188		0.055 (-0.022 to 0.118) 2.096 (1, 35) 0.086	
Stroop interference	Base model^a^	*F*(3, 28) = 2.189, *p* = .112, *R*^*2*^ = 0.190		* F*(3, 36) = 0.199, *p* = .896, *R*^*2*^ = 0.016	
	B (95 %CI)^b^ Fchange (df)^b^ R^2^ ^b^	-0.029 (-0.070 to 0.034) 1.405 (1, 27) 0.230		0.058 (-0.010 to 0.139) 1.704 (1, 35) 0.062	
Letter fluency^1^	Base model^a^	*F*(3, 17) = 2.470, *p* = .097, *R*^*2*^ = 0.304		* F*(3, 26) = 2.215, *p* = .110, *R*^*2*^ = 0.204	
	B (95 %CI)^b^ Fchange (df)^b^ R^2^ ^b^	0.069* (0.001 to 0.120) 4.184 (1, 16) 0.448		0.030 (-0.079 to 0.133) 0.268 (1, 25) 0.212	

^a^ Base model including tumor grade, tumor volume and handedness, without distance as prognostic factor; ^b^Multivariable model, added value for independent variable (distance)

** meaning *p* < .01; * meaning *p* < .05

^1^ Data missing right for *n*=11 and left for *n*=10

For the right-sided structural integrity measures, the minimal mean distance between the tumor and the FAT was a statistically significant prognostic factor for performance on shifting attention and letter fluency. For shifting attention, an extra millimeter in distance led to 0.061 increase (95 %CI 0.025 to 0.109) on the z-score for test performance if other factors in the model remained the same. For letter fluency, an extra millimeter in distance led to an 0.069 increase (95 %CI 0.001 to 0.120) on the z-score of test performance. Other cognitive tests did not show statistically significant results. For the left-sided distance measure, no significant prognostic factors were found for cognitive test performance. Effect sizes (R^2^) ranged from 5 to 30.4 % for the right-sided distance measure, and from 1 to 22 % for left-sided distance measures, accounting for the amount of variation in cognitive test performance.

### Linear regression for structural integrity

Preliminary analyses including visual inspection of scatterplots showed linearity between the structural integrity measures and cognitive test performances. The residues showed homoscedasticity and normality. The results of the linear regression analyses are represented in Table 5, presenting full details of the base model and the multivariable model.

**Table 5:** Linear regression

		Structural measures:			
Cognitive tests:		FA right ^1 × 10e−1^ (*n*=32)	MD right^1 × 10e−4^ (*n*= 32)	FA left ^1 × 10e−1^ (*n*=40)	MD left ^1 × 10e−4^ (*n*=40)
Finger tapping	Base model^a^	*F*(3, 28) = 2.824, *p* = .057, *R*^*2*^ = 0.232		* F*(3, 36) = 0.984, *p* = .411, *R*^*2*^ = 0.076	
	B (95 %CI)^b^ Fchange (df)^b^ R^2^ ^b^	0.123 (-0.52 to 0.75) 0.114 (1, 27) 0.236	-0.092 (-0.395 to 0.296) 0.248 (1, 27) 0.239	0.17 (-0.65 to 1.19) 0.180 (1, 35) 0.081	-0.101 (-0.668 to 0.321) 0.246 (1, 35) 0.082
Symbol digit coding	Base model^a^	*F*(3, 28) = 1.560, *p* = .221, *R*^*2*^ = 0.143		* F*(3, 36) = 0.272, *p* = .845, *R*^*2*^ = 0.022	
	B (95 %CI)^b^ Fchange (df)^b^ R^2^ ^b^	0.497 (-0.013 to 1.007) 3.998 (1, 27) 0.256	-0.233 (-0.445 to 0.015) 3.548 (1, 27) 0.243	-0.010 (-0.48 to 0.50) 0.002 (1, 35) 0.022	-0.044 (-0.308 to 0.158) 0.123 (1, 35) 0.026
Shifting attention	Base model^a^	*F*(3, 28) = 0.767, *p* = .522, *R*^*2*^ = 0.076		* F*(3, 36) = 1.317, *p* = .284, *R*^*2*^ = 0.099	
	B (95 %CI)^b^ Fchange (df)^b^ R^2^ ^b^	0.480 (-0.040 to 0.100) 3.586 (1, 27) 0.184	-0.302* (-0.557 to -0.047) 5.895 (1, 27) 0.242	0.147 (-0.556 to 0.85) 0.181 (1, 35) 0.104	0.036 (-0.322 to 0.393) 0.041 (1, 35) 0.100
Continuous performance	Base model^a^	*F*(3, 28) = 1.343, *p* = .280, *R*^*2*^ = 0.126		* F*(3, 36) = 0.389, *p* = .762, *R*^*2*^ = 0.031	
	B (95 %CI)^b^ Fchange (df)^b^ R^2^ ^b^	0.306 (-0.552 to 1.1641) 0.536 (1, 27) 0.143	-0.335 (-0.756 to 0.086) 2.672 (1, 27) 0.205	-0.230 (-0.716 to 0.256) 0.925 (1, 27) 0.056	0.155 (-0.089 to 0.399) 1.661 (1, 35) 0.075
Stroop interference	Base model^a^	*F*(3, 28) = 2.189, *p* = .112, *R*^*2*^ = 0.190		* F*(3, 36) = 0.199, *p* = .896, *R*^*2*^ = 0.016	
	B (95 %CI)^b^ Fchange (df)^b^ R^2^ ^b^	0.243 (-0.201 to 0.688) 1.264 (1, 27) 0.226	-0.067 (-0.297 to 0.163) 0.361 (1, 27) 0.201	0.207 (-0.362 to 0.776) 0.545 (1, 35) 0.031	-0.178 (-0.462 to 0.107) 1.603 (1, 35) 0.059
Letter fluency^1^	Base model^a^	*F*(3, 17) = 2.470, *p* = .097, *R*^*2*^ = 0.304		* F*(3, 26) = 2.215, *p* = .110, *R*^*2*^ = 0.204	
	B (95 %CI)^b^ Fchange (df)^b^ R^2^ ^b^	0.438 (-0.467 to 1.344) 1.053 (1, 16) 0.347	-0.345 (-0.781 to 0.091) 2.813 (1, 16) 0.408	-0.104 (-0.976 to 0.768) 0.061 (1, 25) 0.205	0.221 (-0.212 to 0.655) 1.104 (1, 25) 0.237

^a^ Base model including tumor grade, tumor volume and handedness, without structural integrity as prognostic factor; ^b^ Multivariable model, added value for each independent variable (structural integrity)

** meaning *p* < .01, * meaning *p* < .05

^1^ Data missing right for *n*=11 and left for *n*=10

For the right-sided structural integrity measures, only MD and not FA was found to be a statistically significant prognostic factor for cognitive test performance on shifting attention. An increase with 1 MD unit led to 0.302 decrease (95 %CI -0.557 to -0.047) on the z-score of test performance if other factors in the model remained the same. For MD and FA the other cognitive tests did not show statistically significant results, nor did they show any effects for the left-sided structural integrity measures. Effect sizes (R^2^) varied from 9 to 41 % for right-sided and 1–24 % for left-sided structural integrity measures, accounting for the amount of variation in cognitive test performance.

## Discussion

Our results suggest that the right FAT is involved in executive functions. Close proximity of a tumor to the right FAT, as measured with tractography, was related to poorer cognitive performance, in particular on tests of shifting attention and verbal fluency. A disturbed microstructural integrity of the FAT, as measured by higher mean diffusivity (MD), was also related to impaired performance on these cognitive tests. To our knowledge, this is one of the first quantitative tractography studies that investigated the function of the right FAT and therefore provides additional evidence for its involvement in executive control functions.

### Shifting attention

Studies in the literature have suggested that the right FAT is involved in functions that require top-down, executive control, in line with results from our study (Aron et al., 2007; Dick et al., 2019). A right hemisphere dominance in attentional systems has been described repeatedly in the last two decades (Petersen & Posner, 2012; Sridharan et al., 2008). According to Petersen & Posner’s theory on the three attentional systems, alerting, orienting and executive control, the latter is responsible for top-down processes. This corresponds to the design of the shifting attention test used in the current study, in which subjects constantly have to respond to changing goals in order to generate the correct response.

Looking more closely at top-down, executive control systems, two separable anatomical networks have been described: the cingulo-opercular control system for maintaining tonic alertness, needed to complete any cognitive task, and the frontoparietal network for moment-to-moment task set adjustment, needed to shift attention (Dosenbach et al., 2008). The seed regions of the FAT, pre-SMA and SMA, are part of the frontoparietal network. Based on this anatomical and functional accordance it can be concluded that the right FAT likely plays an important role in the frontoparietal network. Further evidence for this involvement is found in a study (Shulman et al., 2009), that described participation of the dorsal frontoparietal network in cued endogenous shifts of attention, for which top-down control processes are necessary, and found other regions for stimulus-driven exogenous allocation of attention, necessary for bottom-up processes.

The observation in the current study that the right FAT, but not the left FAT, is involved in the shifting attention task initially appears to contrast with the results from a number of studies that found shifting attention to rely on a left-lateralized frontoparietal network (Brass & Von Cramon, 2002; De Baene et al., 2012; De Baene & Brass, 2013; Dreher & Berman, 2002). However, this difference in results might be best explained by a dissociation between transient and sustained cognitive control in shifting attention tasks and the distinctive brain areas underlying them (Braver et al., 2003). Transient cognitive control comprises processes such as the reconfiguration of a task set or updating of task goals on trials in which the task switches and relies primarily on brain regions in the left hemisphere. Since most task-switching studies examine activity differences between task switches and task repetitions, the left-lateralized frontoparietal network reported in these studies reflects transient cognitive control. Sustained cognitive control, by contrast, comprises the active maintenance of a heightened level of cognitive control over an extended period in situations requiring rapid and flexible alternation between multiple different tasks, and seems to rely primarily on brain regions in the right hemisphere. Since the measure of shifting attention performance in the current study does not dissociate between switch and repeat trials but reflects performance across all trials, this score probably reflects the level of sustained cognitive control instead of transient cognitive control.

While the frontoparietal network also seems to play a role in inhibitory processes (Dodds et al., 2011), the results of the current study suggest that worse performance on shifting attention does not result from inhibition deficits, as performance in inhibition was not affected. This finding is consistent with an article summarizing the cognitive and biological underpinnings of executive functions, which concluded that cognitive flexibility, inhibitory control and working memory, although mutually correlated, can be dissociated as separate executive functions (Miyake & Friedman, 2012). Additionally, in a recent tractography study (Puglisi et al., 2019), the frontal pathways and their role in cognitive control were investigated and an involvement of the FAT in inhibition was also not found (as measured with the Stroop test intra- and postoperatively). Furthermore, this finding is in line with the hypothesis that the right inferior frontal gyrus is not a discrete functional module for inhibition, but may activate or inhibit activities in the right SMA, most likely through the right FAT (Dick et al., 2019). Thus, our findings provide new support that the right FAT plays a prominent role in the frontoparietal network, being responsible for top-down executive control processes that are necessary for shifting attention.

### Letter fluency

Involvement in letter fluency was found for the right FAT, but unexpectedly not for the left FAT. To our knowledge, involvement of the right FAT in letter fluency has not been described before. A likely explanation is that the letter fluency test demands adequate executive functioning. This is supported by a meta-analysis of 30 studies investigating verbal fluency in 1269 traumatic brain injury patients, which concluded that letter fluency tests effectively measure executive functioning. Presumably because this task relies on adequate organization of verbal recollection, as well as on the capacity to initiate, monitor, and inhibit responses (Henry & Crawford, 2004). This explanation is, however, in contradiction with another recent study investigating the underlying cognitive structure of letter fluency (COWAT) in 304 outpatients referred for neuropsychological assessment, which only found a relationship with language components and not with executive functioning components for letter fluency when comparing test results (Whiteside et al., 2016). Therefore, it is still open to debate if this test is a measure of speech and language, executive functioning, or both.

Another explanation for involvement of the right FAT might be that letter fluency requires the ability to shift attention, as repeatedly generating new words that start with the same phoneme involves disengaging attention from one word to be able to relocate the attention to generating a new word. Furthermore, one could imagine that context-monitoring, which is thought to be necessary for shifting attention (Dick et al., 2019), could also be an important underlying process for letter fluency, as the fulfilment of this task relies on the ability to keep a record of the words that have already been generated and of the ones that still need to be generated. Hence, it is likely that performance on the letter fluency test relies in part on similar top-down networks as needed for shifting attention and is therefore related to the right FAT.

We found no involvement of the left FAT in letter fluency. This is in contradiction with earlier clinical studies that found the left FAT to be strongly positively correlated with motor speech functions, among which letter fluency (Birn et al., 2010; Broce et al., 2015; Dick et al., 2019; Kinoshita et al., 2015). Another recent study in healthy individuals, only found a weak correlation between the left FAT and verbal fluency, that did not survive multiple comparison (Vallesi & Babcock, 2020). As in the present study this could have been due to power related issues. Note that our standard clinical neuropsychological test battery (CNS-VS) does not focus on language functions. We have therefore added a verbal fluency test to the clinical protocol. However, in case of time restrictions in the clinical setting, the verbal fluency test was regularly the first to be left out. As a result, the number of subjects with a completed fluency task was a quarter lower than for the other tests. Apart from power-related issues, this might have led to an overestimation of performance for both the right and left FAT, as participants who needed more time for the cognitive tests were more likely to be left out from letter fluency assessment.

### Fractional anisotropy and mean diffusivity

We hypothesized that disturbed structural integrity of the FAT is a negative prognostic factor for cognitive test performance. Remarkably, no effects were found for FA with either of the tests, whereas the effects found for MD were in accordance with the findings of the distance measure, as they were both prognostic factors for letter fluency and shifting attention. While studies in the literature generally find that oedema can affect both FA and MD values (Alexander et al., 2007; Sternberg et al., 2014), compression and infiltration were reported to have opposite effects only on the FA, showing an increase and decrease of FA, respectively (Jellison et al., 2004; Kinoshita et al., 2014; Speckter et al., 2019; Sternberg et al., 2014). When analyzing data at the individual level, results suggested that FA indeed increased when there was significant mass effect on the FAT and decreased when there was mere infiltration by a tumor. This might have obscured possible effects of FA at the group level. As the pattern of compression only occurred in a small number of our patients, we were unable to study this effect with sufficient power and are therefore unable to draw a conclusion. Thus, it appears that only MD, and not FA is a valid measure to represent structural integrity of a white matter tract and link it to functional outcomes in brain tumor patients.

### Clinical implications

Cognitive impairments are often present in glioma patients, and a high incidence is found when measured with neuropsychological tests (Brennum, Engelmann, Thomsen, & Skjøth-Rasmussen, 2018; Rijnen et al., 2019). It is likely that patients who have difficulties with shifting attention and letter fluency on cognitive tests, as found in our study, also experience problems in daily life. Society is full of distractors, and most of our daily activities (e.g. cooking, planning, driving, working) rely on executive control processes. A better understanding of its neurocognitive underpinning also has important implications for neurosurgical practice. In brain tumor surgery, for each patient an optimal balance has to be found between maximal tumor removal (to optimize oncological outcome) and minimal damage to structures that are critical for normal functional performance (to optimize socioprofessional functioning and quality of life) (Duffau & Mandonnet, 2013). Currently, neurosurgeons can use intraoperative electrical stimulation to identify areas or pathways that are critically involved in sensorimotor functions, language and vision (Sanai & Berger, 2018). For executive functions, there are no validated protocols for this purpose yet. Although the present study only enclosed presurgical neuropsychological data and tractography results of lesioned brains, we believe it reveals valuable findings for future research and clinical practice. The anatomo-functional correlations found in the current study, added to the existing evidence from the neurocognitive literature, provides us with leads to guide intraoperative stimulation of the right-sided FAT.

### Limitations and recommendations for future research

The most important limitations concern power-related statistical issues and uncertainties regarding the CSD-based tractography method. A known problem in clinical neuroscience that also impedes this study is the small sample size, which reduces chances to detect or reflect a true effect (Button et al., 2013). The relatively small number of neurosurgical patients with frontal brain tumors, tractography and neuropsychological test results limited statistical power. Still we feel that the conclusions of our study are justified, as both the distance and structural integrity measures pointed in the same direction in this scarce and heterogenic patient group. The other limitations are related to CSD-based tractography. The probabilistic tracking method may generate an unknown number of false positives, even when using prior anatomical knowledge about an existing tract (Maier-Hein et al., 2017). To minimize the influence of false-positive outliers on the overall minimal distance between the FAT and the tumor, distance measures from individual tracts were averaged to create a more reliable and robust parameter. Still, quantifying a spatial relationship between two complex 3D-objects with a single number is challenging. Also, even though the development of the CSD-method has improved tractography greatly by its ability to better resolve regions of crossing fibers, accurate tractography near tumor tissue is still compromised. Therefore, a recommendation for future research would be to use single-shell 3-tissue CSD (SS3T-CSD), as recent evidence shows that this method achieves better tractography results within and close to tumors (Aerts et al., 2019). However, a real ground truth for tractography of white matter tracts and its corresponding measures is still lacking, as is research linking them to functional outcomes.

## Conclusions

In the present study the role of the right FAT was further disentangled by analyzing whether frontal brain tumors that affect the FAT cause changes in executive functions. Although this was an exploratory study, with only presurgical data, combining the spatial and structural measures provided converging evidence that the right FAT is involved in shifting attention and most likely also in letter fluency. Further research is needed to assess whether or not damage to the FAT causes permanent cognitive impairments, and consequently is of importance during neurosurgical procedures.

## Data Availability

Stored in institutional repository, available upon request (incl. lesion overlap map).

## Appendix 1

**Table.6:** Neuropsychological Assessment

Cognitive tests:	Domain score calculation:	Description:
Finger tapping^1^	Taps in 10 s left and right	Press spacebar with index finger as many times as possible in 10 s
Symbol digit coding^1^	Correct responses	Number 1 to 9 refer to different symbols. In 90 s as many numbers as possible have to be matched to the symbols
Shifting attention test^1^	Correct matches False matches	Geometric objects are presented for 2 min and tasks switches to match them either by shape or color.
Continuous performance^1^	Commission errors Omission errors	Respond to a target letter, but not to another letter
Stroop Interference	(Reaction time part 3 - Reaction time part 1) / Reaction time part 1	Part 1: Press spacebar when word is presented Part 3: Press spacebar if color and word meaning do not match
Letter fluency^2^	Total number of correct words	Naming of as many words as possible in 60 s, starting with a specific letter of the alphabet. Repeated 2 times with different starting letters.

^1^ CNS-VS. Derived from “Test-retest reliability and practice effects of a computerized neuropsychological battery: A solution-oriented approach” (Rijnen, van der Linden, Emons, Sitskoorn, & Gehring, 2018)

^2^ Controlled Oral Word Association Test. Derived from “Letterfluency: psychometrische eigenschappen en Nederlandse normen” (Schmand et al., 2008)

## Abbreviations

FAT: frontal aslant tract
IFG: inferior frontal gyrus
SFG: superior frontal gyrus
SMA: supplementary motor area
DW-MRI: diffusion weighted magnetic resonance imaging
FA: fractional anisotropy
MD: mean diffusivity
Mean-min distance: mean minimal distance between each fiber of the tract and the most nearby tumor voxel
CSD: Constrained Spherical Deconvolution

## Code availability

Available upon request.
